# BRAF^V600E^ immunopositive Melanomas Show Low Frequency of Heterogeneity and Association With Epithelioid Tumor Cells: A STROBE-Compliant article

**DOI:** 10.1097/MD.0000000000000285

**Published:** 2014-12-02

**Authors:** Ivana Verlinden, Karin van den Hurk, Ruud Clarijs, Arjan P. Willig, Cecile M.H.A. Stallinga, Guido M.J.M. Roemen, Joost J. van den Oord, Axel zur Hausen, Ernst-Jan M. Speel, Véronique J.L. Winnepenninckx

**Affiliations:** From the Department of Pathology, Maastricht University Medical Centre, Maastricht, The Netherlands (IV, KvdH, CMHAS, GMJMR, AzH, E-JMS, VJLW); GROW-School for Oncology and Developmental Biology, Maastricht University Medical Centre, Maastricht, The Netherlands (KvdH, AzH, E-JMS, VJLW); Department of Clinical Pathology, Atrium Medical Centre Parkstad, Heerlen, The Netherlands (RC); Department of Pathology, St. Laurentius ziekenhuis, Roermond, The Netherlands (APW); and Laboratory of Translational Cell & Tissue Research and Department of Pathology, University Hospital, KULeuven, Leuven, Belgium (JJvdO).

## Abstract

Treatment of BRAF^V600E^-mutant melanoma by small molecule inhibitors that target BRAF or MEK kinases is increasingly used in clinical practice and significantly improve patient outcome. However, patients eventually become resistant and therapeutic improvement is required. Molecular diversity within individual tumors (intratumor heterogeneity) and between tumors within a single patient (intrapatient heterogeneity) poses a significant challenge to precision medicine.

Using immunohistochemistry, we determined the extent of BRAF^V600E^ intratumor and intrapatient heterogeneity and the influence of morphological heterogeneity in a large series of 171 melanomas of 81 patients.

The BRAF^V600E^ mutation rate found in our melanoma series is 44%, with none of 22 (0%) melanoma in situ, 23 of 56 (41%) primary tumors, 28 of 59 (48%) regional metastases, and 24 of 34 (71%) distant metastases harboring the mutation. In general, a diffuse homogeneous immunostaining was seen, even in tumors consisting of more than one cell type, that is, epithelioid, spindle, and/or small cell types. Nevertheless, BRAF^V600E^-mutant melanomas more often had a purely epithelioid cell population (*P* = 0.063), that is more evident among distant metastases (*P* = 0.014). Only two of 75 (3%) mutated specimens (one primary and one metastasis) displayed heterogeneous BRAF^V600E^ expression. The primary tumor was also morphologically heterogeneous and exclusively displayed BRAF^V600E^ in the epithelioid component, confirming an association between BRAF^V600E^ and epithelioid cells. Twenty-eight of 30 patients (93%) had concordant BRAF mutation status between their tumors.

Taken together, BRAF^V600E^ intratumor and intrapatient heterogeneity in melanoma is diminutive, nevertheless, the identified exceptions will have important implications for the clinical management of this disease.

## Abstract 2

Supplemental Digital Content is available in the text

## INTRODUCTION

Cutaneous melanoma is the most aggressive and possibly fatal cutaneous malignancy. When diagnosed early, 95% of melanoma can be cured with radical surgical resection. Advanced melanoma, however, presents one of the most challenging cancers with poor patient outcome.^1^ In addition, treatment options for patients with metastatic melanoma have been very limited. Recent progress in both immunobased and targeted therapies has however revolutionized melanoma treatment, and has shown significant benefit in overall survival of patients with metastatic melanoma.^2^ Especially, the identification that approximately 50% of melanomas harbor a somatic mutation in exon 15 of the *BRAF* oncogene had a significant effect on the treatment of melanoma.^3,4^ BRAF encodes a serine–threonine kinase and is a component of the mitogen-activated protein kinase (MAPK) signaling pathway which is hyperactivated in up to 90% of melanoma cases.^5^ The most common mutation corresponds to a T > A transversion at position 1799, resulting in the substitution of valine by glutamic acid at position 600 of the protein, that is, BRAF^V600E^.^3^ This mutation causes a constitutive activation of the kinase domain of BRAF. The approval of selective BRAF inhibitors, that is, vemurafenib and dabrafenib, and additionally the approval of trametinib, a selective MEK inhibitor, changed the management of metastatic and non-resectable melanoma for patients whose tumors have *BRAF*^*V600*^ mutations.^6,7^ Although these therapeutics can be very effective, unfortunately all patients eventually become resistant.^6,8^ Combination therapy of BRAF and MEK inhibitors was shown to significantly improve progression-free survival but patients still relapse and further improvement of these therapeutics is required.^9^ The clinical detection of *BRAF*-mutant melanoma is currently performed by using a variety of DNA-based molecular techniques, such as direct sequencing, mutation-specific PCR, and mass-spectrometry genotyping.^10–12^ In addition, the immunohistochemical (IHC) detection of the BRAF^V600E^ mutated protein with the use of the BRAF^V600E^ mutant-specific monoclonal antibody, VE1, is gaining interest.^13–19^

Recently, several studies have revealed that tumor heterogeneity poses a significant challenge to precision medicine.^20,21^ Tumor heterogeneity refers to the existence of subpopulations of cells with distinct molecular variation within individual tumors (intratumor heterogeneity) or between tumors of the same histopathological subtype within a patient (intrapatient heterogeneity).^21^ Interestingly, evidence suggests that efforts to predict outcome require the identification of genetically and functionally distinct subclones within a tumor, that is, intratumor heterogeneity, at diagnosis.^22,23^ This indicates that small subclones within a tumor confer primary resistance towards therapy and will expand during therapy leading to tumor progression. Using *BRAF* genotyping techniques, the importance of *BRAF* heterogeneity has drawn attention.^24–27^ Lin et al^24^ showed intratumor heterogeneity of *BRAF*^*V600E*^ in 8 of 10 primary melanomas with the use of a sensitive Mutector assay, as well as by cloning and sequencing of separated alleles. In addition, Yancovitz et al^26^ used laser microdissection and mutation detection via sequencing and BRAF^V600E^-specific SNaPshot analysis to show that in 6 out of 9 primary melanomas there are different proportions of *BRAF*^*V600E*^ and *BRAF* wild-type cells in distinct micro-dissected regions within individual tumors. Lastly, Wilmott et al^27^ reported a case of intratumor BRAF^V600E^ heterogeneity in a melanoma metastasis as determined with real-time PCR and Mass Spectrometric SNP genotyping. In contrast, IHC analyses of BRAF^V600E^ protein with the use of the BRAF^V600E^ mutant-specific monoclonal antibody, VE1, in general, revealed an intense and homogeneous staining of BRAF^V600E^ and hardly any evidence of intratumor and/or intrapatient heterogeneity.^13,14,17–19,28,29^ Moreover, Colombino et al^25^ assessed intrapatient heterogeneity of mutated *BRAF*/*NRAS* and revealed that 84 of 99 (85%) patients who had paired samples of primary and secondary melanomas showed consistent mutation patterns between primary tumors and metastatic lesions. In particular, *BRAF*/*NRAS* mutation frequencies were highly consistent between primary tumor and lymph node (78 of 84 patients [93%]) or visceral metastases (24 of 25 patients [96%]). A significantly less consistent pattern of *BRAF*/*NRAS* mutations rates between primary tumor and brain (16 of 20 patients [80%]) or skin metastases (27 of 36 [75%]) was found, suggesting that in some patients independent subclones are generated. This is in line with research of Yancovitz et al^26^ that showed intrapatient heterogeneity of *BRAF*^*V600E*^ in melanoma metastases in 5 of 19 (26%) patients.

Since it is long known that melanoma consists of distinctive subpopulations of cytologically divergent cells, that is, morphological heterogeneity, the main purpose of the present study was to determine if intratumor morphological heterogeneity correlates with heterogeneous expression of BRAF^V600E^ protein. Moreover, we reasoned that it is of particular interest to identify which tumor cells in the primary lesion have the highest metastatic capabilities and associate them with the presence of mutant BRAF. In addition, BRAF^V600E^ expression was analyzed in patients exhibiting multiple tumors, both primary and metastatic lesions, and we determined the frequency of intrapatient heterogeneity of BRAF^V600E^ mutant expression.

## MATERIALS AND METHODS

### Tumor Material, Histopathologic Analysis, and Clinical Data Collection

This study used tumor tissues (n = 171) from 81 patients (39 male and 42 female; mean age, 58.3 years [age range, 17 to 98 years]) diagnosed between 1995 and 2013 with melanoma in situ (n = 22), primary melanoma (n = 56), regional (skin and lymph node metastases) melanoma metastasis (n = 59), or distant (skin and visceral metastases) melanoma metastasis (n = 34) at the Maastricht University Medical Centre, Maastricht; Atrium Hospital, Heerlen; and the Laurentius Hospital, Roermond, all located in The Netherlands. Patient material was used according to the Code for Proper Secondary Use of Human Tissue (Federation of Medical Scientific Societies, The Netherlands; 2003). Informed consent from patients was not obtained as the data were analyzed anonymously. In total, we collected multiple tumors for 30 patients, including 23 patients with matched primary and metastatic melanomas. Sixteen patients had tissues available from multiple metastatic sites. Haematoxylin & eosin (HE) slides of 149 tumor specimens (excluding melanoma in situ) were reviewed by two pathologists (VW and IV) on the basis of histopathological features according to the most recent World Health Organization classification. Moreover, the percentage of melanoma cells with intracytoplasmic melanin pigment was evaluated and assessment of intratumor cell types was performed according to defined cytomorphological criteria,^30^ that is, (1) epithelioid cell, cells that are round, oval or polygonal with moderate to abundant cytoplasm, the round nucleus is eccentrically located with evenly distributed chromatin; (2) spindle cell, bipolar or dendritic cells with long thin cytoplasmic processes and a centrally placed elongated or ovoid nucleus; (3) small/nevoid cells, round and small monomorphic cells with hardly any cytoplasm, the round nucleus is centrally located and has evenly distributed chromatin. Clinical data and tumor characteristics are given in Table 1.

**TABLE 1:**
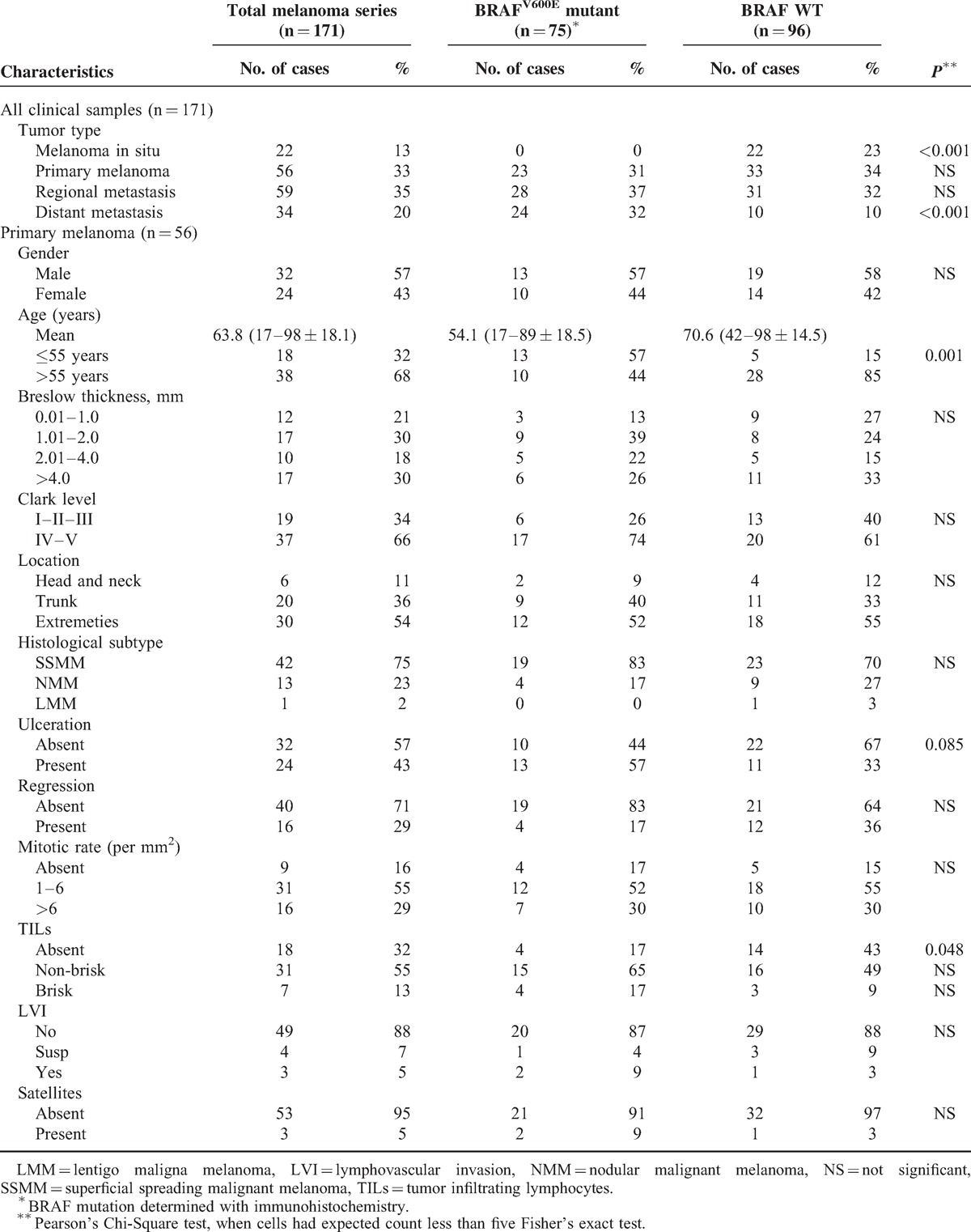
Melanoma Characteristics and Clinical Features

### Genotyping

#### *BRAF* and *NRAS* Pyrosequencing

Forty-five tumor specimens were used to correlate *BRAF*^*V600*^ mutation status as determined by pyrosequencing with IHC staining of BRAF^V600E^ (see below). In addition, 22 cases were analyzed for *NRAS* exon 2 and 3 mutations. For this purpose, three to ten 5–10 μm sections from each formalin-fixed, paraffin-embedded (FFPE) tissue block were subjected to DNA extraction using two different protocols; (1) automated genomic DNA extraction was performed using the Maxwell 16 MDx system with the Maxwell 16 FFPE Plus LEV DNA Purification kit (Promega, Leiden, The Netherlands). In brief, tissue sections were digested three to 16 hours at 70 °C with 180 μL of Incubation Buffer and 20 μL of Proteinase K while shaking. Next, 400 μL of Lysis Buffer was added and the solution was transferred into a cartridge well after which automatic DNA isolation was performed using the Maxwell 16 MDx instrument according to manufacturer's instructions. (2) For DNA isolation with the “raw-extraction” protocol, tissue sections were deparaffinised prior to DNA extraction. Next, 50 to 100 μL TE buffer containing 1% Tween 20-buffer and 6 to 10 μL Proteinase K was added followed by 3 hours incubation at 55 °C. Thereafter, Proteinase K was inactivated by placing the samples 5 to 10 minutes at 95 °C. The tubes were centrifuged (12000 rpm for 1 minute) and the supernatant containing the DNA was transferred to a clean tube. NanoDrop quantification was used to estimate the quality and concentration of extracted DNA (NanoDrop ND-1000 Spectrophotometer). In addition, the grade of DNA fragmentation per sample was estimated using Specimen Control Size Ladder followed by agarose gel electrophoresis.

Pyrosequencing analyses for *BRAF* exon 15 mutations (codon 600), *NRAS* exon 2 (codon 12 and 13) and exon 3 (codon 61) mutations were performed using the PyroMark Q24 MDx system (Qiagen, Venlo, The Netherlands). Target regions were amplified using the Pyromark PCR kit (Qiagen) followed by pyrosequencing analysis on the Pyromark Q24 MDx instrument according to the manufacturer's instructions. For specific primer sequences see Table, Supplemental Digital Content 1, http://links.lww.com/MD/A98, and for assay conditions see Table, Supplemental Digital Content 2, http://links.lww.com/MD/A98. Sequence analysis was performed using the PyroMark Q24 2.0.6 software (Qiagen).

#### BRAF Immunohistochemical Analysis

From 171 tumor specimens, 3-μm thick FFPE sections were freshly cut, mounted on microscopic slides ([K8020] Dako, Glostrup, Denmark) and air dried at 65 °C for 30 minutes. BRAF IHC analysis was done on a Dako Autostainer Link 48 system. In brief, antigen retrieval was performed with EDTA (pH 9) using the PT link (Dako) for 10 minutes at 97 °C, subsequently followed by 5 minutes blocking with EnVision FLEX Peroxidase-Blocking Reagent, 20-minutes primary antibody incubation with “Mouse Anti-Human BRAFV600E Monoclonal Antibody VE1 (E19292—Spring Bioscience [1:50 dilution]), 15 minutes incubation with EnVision FLEX + Mouse (Linker) and 20 minutes incubation with Envision FLEX HRP-labeled polymer. Visualization was performed using chromogen substrate, either DAB for 10 minutes or AEC (for heavily pigmented tumors) for 20 minutes and tissues were counterstained with hematoxylin. A mutant control (*BRAF* c.1799 T > A [p. V600E]) as determined by pyrosequencing and a wild-type control (*BRAF* wild type as determined by pyrosequencing) were included in each staining procedure.

#### IHC Interpretation

All immunostained slides were evaluated by two pathologists (VW and IV) blinded to all clinical, histopathological, and mutation data. The VE1 staining was scored positive when there was clear cytoplasmic staining in the tumor cells. Faint diffuse staining, finely granular or coarsely clumped cytoplasmic staining, nuclear staining, no staining or weak staining of single cells was scored as negative.

### Statistical Analyses

The Pearson's chi-square (χ^2^) test was used to see if there is a correlation between *BRAF* mutation status as determined by pyrosequencing and VE1 expression. Similarly, the Pearson's chi-square test was used to determine an association between VE1 expression and various tumor characteristics, if cells had an expected count of less than 5; the Fisher's exact test was applied. All statistical analyses were two-sided, and *P* < 0.05 was considered statistically significant. All analyses were done with the statistical package IBM SPSS Statistics 21.

## Results

### Melanoma Characterization and Morphological Tumor Heterogeneity

To determine the extent of morphological tumor heterogeneity in our melanoma series, HE stained slides of 149 tumor specimens, including 56 primary melanomas, 59 regional metastases, and 34 distant metastases, were reviewed. In addition, primary tumors were reviewed in detail for histopathological and clinical characteristics, including Breslow thickness, Clark level, location, histological subtype, ulceration, regression, mitotic rate (per mm^2^), presence of tumor infiltrating lymphocytes (TILs), lymphovascular invasion (LVI), presence of microsatellites, and the presence of an adjacent nevus (Table 1). In most cases, melanomas showed a purely epithelioid cell population (118 of 149 cases (79%]) (Table 2). Our series contained only two tumors, one primary and one distant metastasis, obtained from two patients, that displayed a purely spindle cell population. Additionally, one distant metastatic sample contained purely small cells. Twenty-eight of 149 (19%) cases displayed morphological intratumor heterogeneity meaning that more than one cell type within the same tumor was observed (Table 2). In cases with a mixed cell population, epithelioid cells were always present, and in 21 of 28 (75%) cases, this was the major cell component (≥50% of cells). Interestingly, primary melanomas more often (*P* < 0.001) had a mixed cell population (19 of 56 [34%]) and, hence, are morphologically more heterogeneous compared with regional (4 of 59 [7%]) and distant (5 of 34 [15%]) metastases (Table 2). Additionally, we determined the grade of pigmentation and found that 25 of 149 (17%) cases contained melanin in more than 80% of the tumor cells. Twenty-one (84%) of these cases had a purely epithelioid cell population, and the other four cases had ≥75% of epithelioid tumor cells. Particularly regional metastases were often heavily pigmented (17 of 59 [29%]) when compared with primary melanomas (6 of 56 [11%]) and distant metastases (2 of 34 [6%]) (*P* = 0.001; Table 2). Moreover, these tumors most often had a purely epithelioid cell population, that is, 55 of 59 (93%), when compared with primary melanoma (36 of 56 [64%]) and distant metastases (27 of 34 [79%]) (*P* < 0.001; Table 2).

**TABLE 2:**
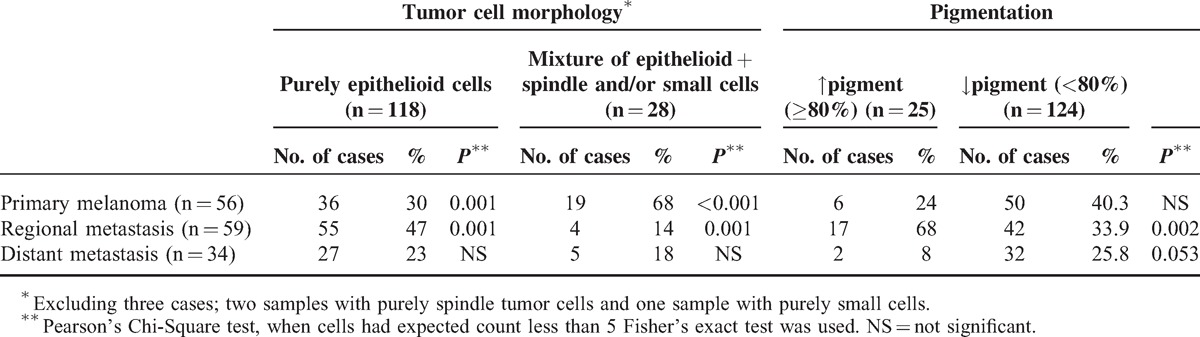
Tumor Cell Morphology and Pigmentation of Primary Melanomas, Regional Metastases and Distant Metastases

Next, we examined morphological tumor heterogeneity between paired primary and/or metastatic tissues within the same patient. In 20 of 30 (67%) patients with paired samples the composition of cell population (22 of 30 patients [73%]) and percentage of melanin pigmentation (22 of 30 patients [73%]) were similar. If not, in the majority of cases, patients with a primary tumor consisting of mixed cell types harbored metastases displaying exclusively epithelioid cells. Moreover, most metastatic tumors had a higher percentage of pigmented cells when compared with the primary tumor (see Table, Supplemental Digital Content 3, http://links.lww.com/MD/A98, providing results of intrapatient heterogeneity of cell morphology and pigmentation in patients).

### High Correlation Between *BRAF*^*V600E*^ Mutation Status and Immunopositivity for the Mutated Protein

**FIGURE 1:**
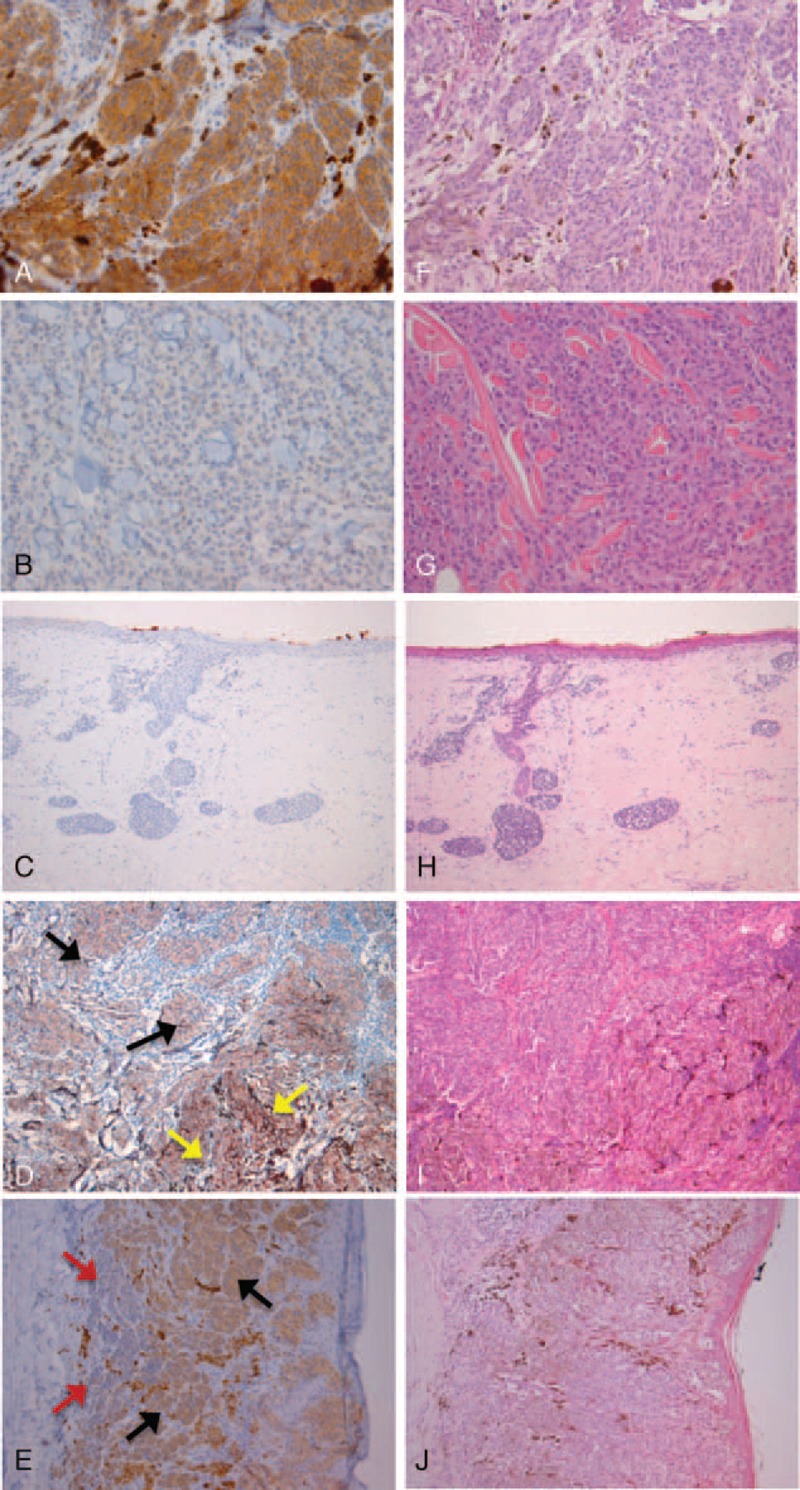
(A and F) melanoma, mutated for BRAF^V600E^ in purely epithelioid background. (A) Diffuse homogeneous immunostaining with VE1. (F) Corresponding HE staining. (B and G) melanoma, wild-type for BRAF^V600E^ in purely epithelioid background. (B) Negative immunostaining with VE1. (G) Corresponding HE staining. (C and H) melanoma, discordant case with tumor regression. (C) negative immunostaining with VE1. (H) Corresponding HE staining. (D and I) melanoma, mutant BRAF^V600E^. (D) Diffuse homogeneous immunostaining with VE1 positive epithelioid (black arrows) and spindle (yellow arrows) cells. (I) Corresponding HE staining. (E and J) melanoma with heterogenic BRAF^V600E^ expression. (E) Heterogeneous immunostaining with VE1 positive epithelioid (black arrows) cells and VE1 negative small (red arrows) cells. (J) Corresponding HE staining.

To be able to correlate BRAF-mutant protein expression with tumor characteristics and morphological tumor heterogeneity, we first determined the correlation between BRAF^V600E^-mutant immunopositivity assessed with the specific monoclonal antibody VE1 and the presence of the mutation as determined with pyrosequencing in 45 out of 149 randomly selected tumor samples. Twenty-nine of 45 (64%) tissues harbored the *BRAF*^*V600E*^ genotype and no other *BRAF* codon 600 mutations were identified (Table 3). The same tissues were subsequently immunostained for BRAF^V600E^ protein and a high concordance between *BRAF*^V600E^ genotype and VE1 expression was observed (*P* < 0.001; staining sensitivity 98% and specificity 100%). Twenty-eight of 45 (62%) tissues were stained positive for the mutation (Figure 1A; Table 3). All 16 *BRAF* wild-type specimens lacked BRAF^V600E^ expression as determined with IHC demonstrating 100% specificity of the staining (Figure 1B; Table 3). Importantly, in cases sequenced as wild type, we never observed even single VE1-positive cells. The single discordant tissue was positive for the gene mutation, however, no BRAF^V600E^ expression was detected (Figure 1C). Also after re-testing, the pyrosequencing result remained positive and IHC negative. Notably, this specimen showed abundant tumor regression.

**TABLE 3:**
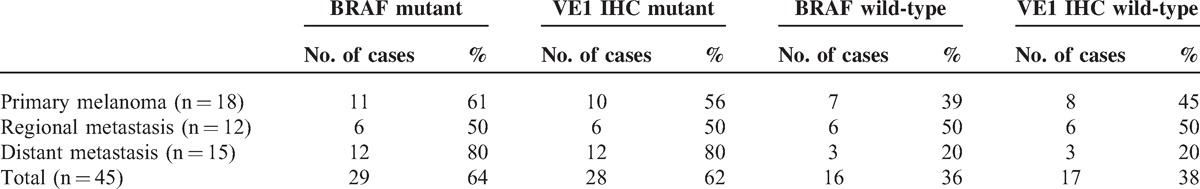
Correlation Between BRAF^V600E^ gene mutation status and immunopositivity (VE1 IHC) for the mutated protein

### Distribution of BRAF^V600E^ and Correlation With Melanoma Characteristics and Clinical Features

Next, we immunostained the additional 104 tumor specimens with VE1, and also included 22 in situ melanomas, hence, together with previously stained 45 specimens we obtained data on BRAF-mutant expression of 171 tissues (Table 1). Primary melanomas displayed BRAF-mutant protein expression in 23 of 56 (41%) specimens and, for comparison, in 23 of 45 (51%) patients; 28 of 59 (48%) regional metastases or 13 of 25 (52%) patients had BRAF^V600E^ expression, and the highest proportion of BRAF^V600E^ (*P* < 0.001) was detected among distant metastases, that is, 24 of 34 (71%) tumors or 12 of 18 (67%) patients. Intriguingly, none of 22 in situ melanomas had BRAF^V600E^ expression (*P* < 0.001). Moreover, BRAF^V600E^-mutant primary melanoma inversely correlated with age (*P* = 0.001) and correlated with the presence of TILs (*P* = 0.048; Table 1). Our series contained two primary melanomas with an adjacent nevus and both melanoma and nevus cells lacked immunoreactivity for the V600-antibody.

### Correlation of BRAF^V600E^ Expression With Morphological Tumor Heterogeneity

We subsequently compared VE1 protein expression with tumor cell morphology and pigmentation (Table 4). Interestingly, BRAF^V600E^-mutant melanoma most often harbored a purely epithelioid cell population (64 of 75 [85%]) (*P* = 0.063); this is more evident in the distant metastases subgroup (*P* = 0.014). Also, a trend towards an association between a mixed cell population and the BRAF wild-type phenotype was observed (18 of 28 [64%]) (*P* = 0.086), which reached statistical significance among distant metastases (*P* = 0.019). No association between pigmentation and the BRAF mutant phenotype was found (Table 4). Notably, off all 28 tumors with a mixed cell population, the epithelioid component represents at least half of the cell population in 21 (75%) cases and 9 of these 21 (43%) cases are BRAF mutant. The remaining 7 cases had an epithelioid component that was less than half of the cell population and only 1 of 7 (14%) cases displayed mutant BRAF expression. This data together postulate that BRAF^V600E^ mutation is associated with epithelioid tumor cells.

**TABLE 4:**
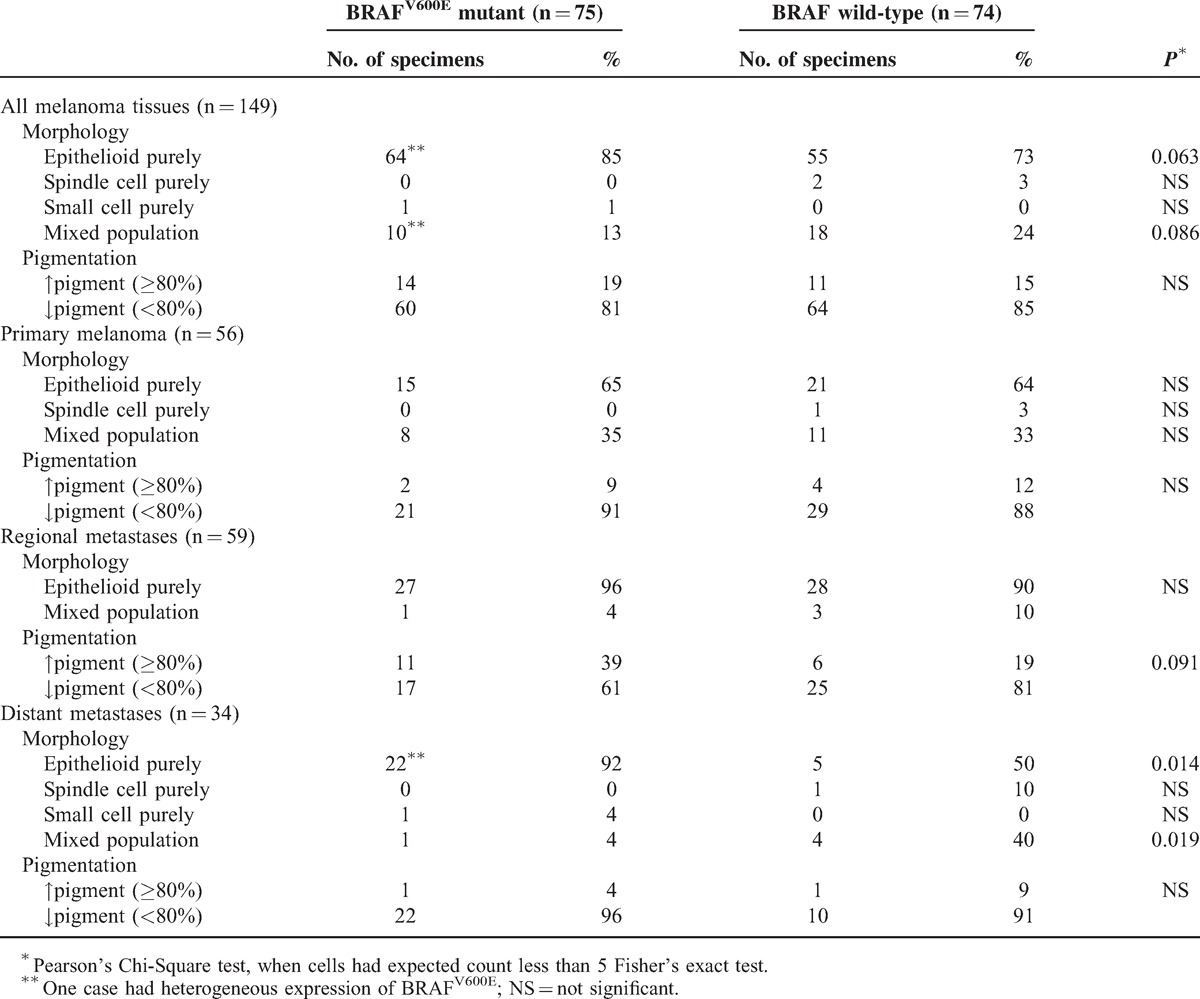
Histological Appearances of Primary, Regional, and Distant Metastatic Melanoma Tissues According to BRAF Mutation Status

### Intratumor and Intrapatient Heterogeneity of BRAF^V600E^ Protein Expression

In general, VE1 immunostaining was homogeneously positive (n = 73) (Figure 1A and 1D) or negative (n = 96) (Figure 1B) except for two tumor samples, that is, one primary tumor and a paired metastasis, showing heterogeneous expression of BRAF^V600E^. Interestingly, the primary melanoma that displayed intratumor heterogeneity for BRAF^V600E^ was in addition morphologically heterogeneous and the mutation was exclusively displayed in the epithelioid component while the small cell component was negative (Figure 1E). This further strengthens our previous observation that BRAF^V600E^ mutation is associated with the epithelioid cell component. The paired metastasis also displayed heterogeneous VE1 expression in a purely epithelioid background.

As we had paired tumor samples for 30 patients, we were able to examine intrapatient heterogeneity of mutant BRAF as determined with both pyrosequencing and IHC, and in some cases *NRAS* mutation as determined with pyrosequencing. All but two patients (28 of 30 [93%]) had concordant BRAF mutation status between their tumors. One patient had a primary tumor that was *NRAS* wild type and *BRAF* mutant and stained homogeneously positive with VE1. Nevertheless, the paired lymph node metastasis stained negative for BRAF^V600E^ and confirmed to be *BRAF* wild type. Unexpectedly, this tumor harbored a *NRAS* mutation. Moreover, the primary tumor of this patient contained a 20% pigmented, purely epithelioid cell population whereas the lymph node metastasis of the same patient had a mixed population of epithelioid (95%) and small/nevoid cells (5%) that were 100% pigmented. The other patient had a discordant BRAF mutation status between two regional metastases, that is, a lymph node metastasis that did not display BRAF^V600E^ and a skin metastasis with the mutation. This patient shared the same histological features, that is, a pure population of epithelioid tumor cells that were not pigmented, in both metastases.

## DISCUSSION

Cutaneous melanoma represents one of the most aggressive cancers and a major challenge for the medical oncologist. The arrival of the targeted therapy revolution has led to significant improvement in melanoma treatment. However, therapeutic resistance and adverse effects to therapies underscore the importance to clarify the pathobiology of melanoma, which ultimately leads to an enhanced molecular-based medical approach. The goal of this study was to determine the extent of BRAF^V600E^ intratumor and intrapatient heterogeneity and the influence of morphological heterogeneity in a large series of 171 melanomas belonging to 81 patients.

Cutaneous melanoma is a morphologically heterogeneous malignancy with different histological appearances within one single tumor.^30^ By analyzing tumor cell type and the presence of intracytoplasmic pigment (as signs of melanocytic differentiation) within single tumors we observed that primary melanoma specimens more often (34% [*P* = 0.001]) had a mixed cell population compared with metastatic tissues (10%). Morphological plasticity is necessary for tumor cells to assume a shape that is suitable for migration and invasion.^31^ Hence, the observation that primary melanomas are morphologically more heterogeneous is in line with the idea that these tumor cells endeavor survival and formation of (distant) metastases. Regional metastases generally had a purely epithelioid cell population (93% [*P* = 0.001]) and these tumors are, compared with primary melanomas (11%) and distant metastases (6%), more often heavily pigmented (29% [*P* = 0.001]). Concerning intrapatient morphological heterogeneity, two thirds of paired cases were comparable in terms of pigmentation and cell population. In the remaining third, the epithelioid and heavily pigmented cells were more often present in the metastases suggesting that these cells might have the highest metastatic potential. This assumption is in line with the observation that epithelioid cell melanomas have greater DNA ploidy abnormalities than spindle cell melanomas.^32^ Moreover, epithelioid cell melanomas were found to be a prognostic factor of poor response to immunological treatment.^33^ Interestingly, the purely epithelioid cell component had the highest prevalence in both primary (64%) and metastatic (88%) melanomas and only 2% of cases did not contain any epithelioid cells. The observation that the majority of cells in the primary tumor are likely to have a high metastatic potential, that is, epithelioid cells, might partly explain why this tumor is very aggressive and able to metastasize quickly.

We reasoned that the presence of distinctive cell populations might underlie a different genetic background or BRAF mutation status as well. This is potentially important in understanding the commonly observed therapeutic resistance to BRAF inhibitors of these tumors. Contradictory statements about the heterogeneity of *BRAF* mutation status in primary and metastatic melanomas exist.^13,14,17–19,24–28^ Most studies using IHC detection of BRAF^V600E^ hardly found any evidence of intratumor heterogeneity and in general most BRAF^V600E^ melanomas stained intensely and homogeneously.^14,18,19,28,29^ Wilmott et al^13^ reported a heterogeneous immunoreaction in 13 of 58 (22%) cases. Studies of heterogeneity that analyzed the *BRAF* genotype in a small number of cases suggest that the majority of melanomas contain both wild-type and mutant BRAF cells.^24,26,27^ In the present study, we virtually did not observe heterogeneous expression of BRAF^V600E^ at the single-cell level. In BRAF wild-type melanomas, we never identified a minor positive subpopulation and BRAF-mutant protein expression was homogeneous in most cases (97%), also when tumors harbored a mixed cell population. This demonstrates that the mutation is most likely a clonal event in cutaneous melanoma and would imply that the alteration into different tumor cell morphologies occurs at a later stage in time, that is, after the BRAF mutation is acquired, for example, by epigenetic mechanisms. It has for instance been reported that the epithelioid melanoma cell type (versus all other cell types) is the most powerful independent predictor of both *RASSF1A* and *p16* promoter hypermethylation.^34^ Interestingly, it was observed that BRAF^V600E^-mutant melanoma more often harbored a purely epithelioid cell population that was particularly evident in the distant metastases subgroup. Conversely, an association between a mixed cell population and BRAF wild-type phenotype was observed, which reached statistical significance among distant melanomas. No association between pigmentation and the BRAF mutant phenotype was detected. The fact that the BRAF mutation was found to be associated with epithelioid cells, that is, cells that potentially have the greatest metastatic capability, might be important to better understand the biologically mechanism that underlie mutant BRAF and melanoma. It has previously been shown that the presence of larger, rounder, that is, epihelioid cells, and in addition more pigmented tumor cells were distinguishing features of melanomas with BRAF mutation.^35^ Our series only contained two melanoma samples, belonging to the same patient, which had heterogeneous expression of BRAF^V600E^. The primary melanoma exclusively displayed the mutation in the epithelioid component while the small cell component was negative, again confirming the association between BRAF^V600E^ and epithelioid tumor cells. A paired metastasis had variable VE1 expression within a purely epithelioid cell population, that is, with obvious positive and negative cells. In contrast, another distant metastasis from the same patient displayed exclusively BRAF^V600E^ mutant epithelioid cells. Intriguingly, this patient initially responded well to vemurafenib treatment but relapsed within a few weeks. Autopsy material of this patient showed distinctive BRAF mutant subclones with evidently stronger BRAF^V600E^ expression compared to the rest of the tumor tissue.

As multiple tumors within a patient have been shown to respond heterogeneously to BRAF inhibitor treatment,^36^ we also determined the level of intrapatient heterogeneity of BRAF^V600E^ in our series. Previous research has shown that 4% to 25% of melanoma patients have heterogeneous *BRAF*^*V600E*^ genotype between their tumors depending on the type of metastasis.^25,26^ Our study contained 2 (7%) patients with discordant BRAF mutation status between paired tumors. That is, one patient had a primary tumor that displayed BRAF^V600E^ expression whereas the paired metastasis contained solely BRAF wild-type tumor cells, but harbored a *NRAS* mutation. The most likely explanation is that this patient had a second primary tumor that did metastasize to the lymph node. The other patient had a discordant BRAF mutation status between two regional metastases, that is, a BRAF wild-type lymph node metastasis and a BRAF-mutant skin metastasis. These patients illustrate clinical treatment difficulties as the presence of molecular variation between tumors within single patients would entail different treatments to eradicate all individual tumors.

The BRAF^V600E^ mutation rate found in our melanoma series is 44% and increases with melanoma progression, that is, none of melanoma in situ, 41% of primary melanomas, 48% of regional metastases, and 71% of distant metastases displayed the mutation. Several groups have reported a strong inverse correlation between age and *BRAF* mutation prevalence,^37,38^ and within our primary melanoma subgroup we also observed a significantly higher BRAF mutation rate among young patients (≤55 years). In addition, a significant association between the presence of BRAF^V600E^ and TILs was observed which is in agreement with current literature and further supports the observation that BRAF^V600E^ initiates an immune reaction to the primary melanoma in vivo.^39,40^

The observation that all in situ melanomas were BRAF wild type is intriguing. It is known that mutant BRAF protein induces cellular senescence (oncogene-induced senescence) by increasing the expression of p16^INK4a^ in healthy melanocytes.^41^ Therefore, most BRAF-mutant nevi never transform to malignant melanoma. We reasoned that the lack of BRAF mutation in early melanoma in situ prevents these tumor cells to go into senescence, thus maintaining malignant potential. During tumor progression, after cells underwent other/additional molecular changes, the BRAF mutation is acquired or the amount of mutated protein increases in numerous cells leading to increased tumor growth and metastasis formation. This is in line with research performed by Dong et al^42^ that showed a high frequency (62%–72%) of *BRAF* mutations in melanocytic nevi, vertical growth phase (VGP) melanomas, and metastatic melanomas, whereas *BRAF* mutations were only detected in 10% of the earliest stage or radial growth phase (RGP) melanomas.

The BRAF^V600E^ mutation detected by pyrosequencing was almost perfectly predicted by immunostaining with the mutation-specific anti-BRAF^V600E^ antibody (VE1), thereby confirming previous studies.^14–16,18^ Only one tumor tissue carried the *BRAF*^*V600E*^ genotype but did not display mutated protein expression (sensitivity of 98%; sensitivity of 100%). The discordant tumor tissue showed abundant tumor regression which might have interfered with the result. It has been reported that the antigenicity of the VE1 epitope is affected by tissue coagulation or early necrosis.^14,18^ Taken together, the immunohistochemical detection of BRAF^V600E^ expression seems to be a rapid and accurate method for detection of the *BRAF*^*V600E*^ mutation and might be generally applied in routine clinical diagnostics.

In summary, our data show that primary melanomas are morphologically more heterogeneous than melanoma metastases and that the epithelioid (pigmented) tumor cell potentially has the greatest metastatic capacity. This study demonstrates that the BRAF mutation is associated with the epithelioid cell type. In general, BRAF mutated protein is present in all tumor cells indicating that this genetic aberration is a common clonal event in melanoma. Intratumor and intrapatient heterogeneity of BRAF^V600E^ is very rare; however, few exceptions give emphasis to treatment difficulties as differences in the genetic landscape require different treatment.
