# BRAF^V600E^ Immunopositive Melanomas Show Low Frequency of Heterogeneity and Association With Epithelioid Tumor Cells: A STROBE-Compliant Article: Erratum

**DOI:** 10.1097/01.md.0000464339.19779.8f

**Published:** 2015-04-10

In the article “BRAF^V600E^ Immunopositive Melanomas Show Low Frequency of Heterogeneity and Association With Epithelioid Tumor Cells: A STROBE-Compliant Article”,^1^ which appeared in Volume 93, Issue 28 of *Medicine*, several corrections have been made to Tables 2 and 4.

In addition, the sentence “The Pearson's chi-square (χ^2^) test was used to see if there is a correlation between BRAF mutation status as determined by pyrosequencing and VE1 expression” should have read “The Cohen's kappa (k) coefficient was used to see if there is a correlation between BRAF mutation status as determined by pyrosequencing and VE1 expression” in the Statistical Analyses section.

A k coefficient value has also been added to the Results section.

The article has since been corrected online.
